# The Effect of Sleep on the Health and Dietary Behaviours of GAA Athletes

**DOI:** 10.3390/nu16111660

**Published:** 2024-05-28

**Authors:** Matt Moran, Lisa Ryan, Rónán Doherty, Michelle Biggins, Karen M. Keane

**Affiliations:** 1 Department of Sport, Exercise and Nutrition, Atlantic Technological University, H91 T8NW Galway, Irelandlisa.ryan@atu.ie (L.R.); 2 Sports Lab North West, Atlantic Technological University Donegal, Letterkenny Campus, Port Road, F92 FC93 Letterkenny, Ireland; ronan.doherty@atu.ie; 3 Sport Ireland Institute, National Sport Campus, Abbotstown, D15 Y52H Dublin, Ireland

**Keywords:** sleep, health, GAA athlete, food cravings, dietary behaviours

## Abstract

Decreased sleep quality and duration is associated with an array of negative health outcomes. Evidence suggests athletes are susceptible to sleep inadequacies that may in turn affect their health and dietary behaviours. This study aimed to explore the sleep profile of both male and female Gaelic games players, at an elite and sub-elite level and compare how poor sleep relates to subjective health complaints and food cravings. One hundred and seventy Gaelic games players completed the Pittsburgh Sleep Quality Index (PSQI), Subjective Health Complaints Inventory (SHC) and Food Cravings Questionnaire-Trait-Reduced (FCQ-T-r). Participants were categorised into two groups: poor sleepers (PSQI ≥ 5) and good sleepers (PSQI < 5). Outcome measures of health and food cravings were analysed across the groups, Mann–Whitney U tests were used to assess differences, and Spearman’s rank-order correlations were used to determine relationships between variables. Sixty-seven % of athletes were categorised as poor sleepers. There were no significant differences in PSQI scores across genders (*p* = 0.088) or playing level (*p* = 0.072). Poor sleepers experienced significantly increased SHC (*p* < 0.001) and female athletes had significantly more SHC compared to males (*p* < 0.001). Female athletes experienced more food cravings than males (*p* = 0.013). However, there were no significant differences in food cravings between good and poor sleepers (*p* = 0.104). The findings suggest a high prevalence of poor sleepers amongst GAA athletes. Furthermore, a significant relationship exists between poor sleep and health complaints with females at a higher risk of worsened health complaints and higher food cravings. Sleep screening and education interventions to enhance sleep in GAA athletes are advocated.

## 1. Introduction

Sleep is an indispensable component of human health and well-being [1]. It is one of the leading lifestyle behaviours that is adversely associated with all-cause mortality, cardiovascular disease and cancer [2]. Decreased sleep quality and duration is also associated with an increased incidence of chronic health disorders including obesity and type 2 diabetes as well as an array of other negative health outcomes [3,4]. Above all, sleep is required for the survival and function of every human being. The estimated ‘basal sleep need’ for adults is 7–8 h [5].

Sleep has been identified as a key factor in an athlete’s ability to train, maximise their training response, recover and perform [5]. In addition, improved sleep duration and quality is associated with a higher chance of competitive success in team sport [1,6]. However, evidence indicates that elite athletes are particularly susceptible to sleep inadequacies characterised by habitual short sleep duration (<7 h) and poor sleep quality [7,8]. This may be attributed to poor awareness with respect to their specific sleep needs [9]. It has been reported that athletes are particularly susceptible to sleep inadequacies due to heavy training volume/load, busy schedules, late-night training and competition and travel all of which may have a negative effect on sleep [1,8].

Across sports, one of the leading determinants of athletic success is training availability. It is important to note that an athlete’s health, which includes injuries and illnesses, poses the most significant barrier to an athlete’s training availability [10], with a strong relationship between time lost to injury and illness in elite athletes and their subsequent performance outcome demonstrated. Training interruptions due to illness or injury are associated with a significantly lower chance of achieving predefined performance goals highlighting the importance of keeping athletes fit and healthy [5]. Poor sleep (<7 h) has been associated with lower general health, immunosuppression and increased susceptibility to upper respiratory infections in the general population [1]. However, studies exploring the relationship between sleep and general health in athletes are lacking [3].

Sleep may encourage overconsumption of hyperpalatable foods, and thus provide support for links between obesity and sleep [11]. For athletes, positive adaptation to training can be optimised through effective nutritional strategy and recovery. Hence, to meet the demands of an athlete’s training objectives, nutritional needs must be adapted accordingly [12]. Poor sleep is associated with increased catabolic and reduced anabolic hormones, which results in impaired muscle protein synthesis subsequently blunting training adaptations and impacting recovery [12]. Chronically, athletes may develop an altered glucose metabolism and neuroendocrine function, causing concern regarding carbohydrate metabolism, appetite, food intake, and protein synthesis. These factors can all influence an athlete’s nutritional, metabolic, and endocrine status negatively and hence are liable to reduce athletic performance [7].

The Gaelic Athletic Association (GAA) is an amateur sporting organisation that embodies the most prominent sports in Ireland, Gaelic football, hurling and camogie [5]. GAA clubs are the cornerstone of the GAA, developing sub-elite players of all ages. The most proficient club-level players are presented with the opportunity to represent their respective county, where they engage in maximal training within the norms of GAA and compete at the highest possible level in their sport. Thus, these athletes are considered elite [13,14]. In Gaelic football, hurling and camogie, the team consists of 15 players with each team having a goalkeeper, two lines of three defensive players (full-back and half-back), two midfielders, and two lines of attacking players (half forward and full forward). The principles of play of these games are to disrupt the defence by striking the Gaelic football or the solid leather slíotar [slit er] (hurling/camogie) through the opposition’s goalposts, either below the crossbar for a goal (worth three points) or above for a point. These field-based multi-directional sports are intermittent in nature and physiologically demand repeated bouts of high-intensity, anaerobic activity intertwined with sustained periods of aerobic activity [15]. Additional demands include a variety of offensive and defensive skills which involve evasion, high running velocities, agility, strength and power [13,15].

Evidence pertaining to sleep and GAA players is limited to a single study. A study conducted in 2018 showed that a remarkable 48% of the population studied were classified as poor sleepers. Furthermore, those classified as poor sleepers experienced significantly increased subjective health complaints (SHC) [5]. However, this study included elite male GAA players only. Interestingly, gender has been identified as a risk factor for sleep problems in athletes, with a greater incidence of sleep problems in female athletes [16]. Therefore, this appears to be a considerable oversight given the prominence of female participation in the GAA and the considerable impact sleep may have on health, performance and dietary behaviours [7]. The aims of this study are the following:

1. To explore the sleep profiles of male and female, elite and sub-elite GAA players;
2. To investigate the relationship between sleep and general health in GAA players;
3. To investigate the relationship between sleep and food cravings in GAA players.

The authors hypothesise that there will be a high prevalence of poor sleep amongst GAA players. It is also hypothesised that poor sleep will be linked to increased health complaints and greater food cravings in this population.

## 2. Materials and Methods

### 2.1. Design

An observational, cross-sectional study was conducted. Snowball and voluntary response sampling were employed to recruit participants. All participants provided written informed consent prior to data collection. The participants were then provided with an online link to the questionnaire battery via Microsoft Forms. All participants completed demographic data before completing sleep, health and nutrition questionnaires. Participant recorded their sex, age, body mass, height, sport, athlete type (elite or sub-elite) and phase of season (pre-season, competition, or off-season). All procedures were approved by the local research ethics committee (ID: 05012023) in accordance with the Declaration of Helsinki.

### 2.2. Participants

To be included in this study, participants must play GAA (Gaelic football, hurling and/or camogie) at a competitive level and be over the age of 18 years old. Participants who played GAA recreationally or those < 18 years old were excluded from the study. A sample size of *n* = 128 was statistically estimated prior to recruitment using a priori analysis in G*Power with an effect size of 0.5.

A total of 170 GAA athletes (105 males; 65 females) participated in the study. Of participants, 62% played Gaelic football, 12% hurling/camogie and 26% played both. Moreover, *n* = 57 (*n* = 17 female; *n* = 40 male) played at senior intercounty level and were considered elite in their sport while *n* = 113 (*n* = 48 female; *n* = 65 male) were club-level players who were classified as sub-elite [14]. Participants also categorised themselves as in competition (39%), off-season (56%) and pre-season (5%). Further participant characteristics are displayed in Table 1.

### 2.3. Subjective Health Complaints Inventory (SHC)

The SHC inventory was employed to assess the general health of these amateur athletes. This questionnaire identifies common health complaints and is considered a comprehensive indicator of general health [3]. The questionnaire comprises 29 issues pertaining to subjective somatic and psychological complaints encountered over the preceding 30 days [17]. Some of the health complaints included were depression, anxiety, chest pain, dizziness, stomach discomfort [3]. Participants were asked to rate each complaint on a four-point scale based on the severity experienced (0 = none, 1 = some, 2 = much, 3 = severe). A total score was obtained for each athlete, higher scores were representative of lower general health [3]. The SHC has produced a Cronbach’s alpha of 0.82 for women and 0.75 for men, demonstrating its internal consistency [17].

### 2.4. Pittsburgh Sleep Quality Index (PSQI)

Sleep was assessed using the Pittsburgh Sleep Quality Index (PSQI), which assesses subjective sleep quality over the previous month. This standardised, self-reported sleep questionnaire presented several subscale measures to participants, including sleep quality, sleep latency, sleep duration, habitual sleep efficiency, sleep disturbances, use of sleep medication, and daytime dysfunction. The sum of these subscale scores formulated an outcome measure (0–21) synthesising sleep quality and quantity, referred to as a global score. Higher scores indicated poorer sleep quality. This questionnaire classifies good sleepers as <5 and poor sleepers as ≥5, using the PSQI. The PSQI has demonstrated sufficient internal homogeneity, reliability and validity [18]. With the adoption of a cut-off score of 5, the PSQI has a sensitivity of 89.6% and specificity of 86.5% for identifying sleep disorders [5]. Additionally, the PSQI has established a high and significant correlation with sleep log data (*r* = 0.81 for sleep duration and *r* = 0.71 for sleep onset latency) [5,19].

### 2.5. Food Cravings Questionnaire-Trait-Reduced (FCQ-T-r)

Food cravings were assessed using the Food Cravings Questionnaire-Trait-Reduced (FCQ-T-r). The FCQ-T-r establishes a self-reported measure that is concise, valid and reliable [20]. Participants rated a series of prompts based on their individual experiences. A 6-point scale was employed, which extended from never to always. An objective outcome score was obtained. Higher scores are associated with individuals who suffer from a range of unfavourable health outcomes such as bulimia nervosa, binge eating disorder, obesity, a positive correlation with higher body mass index, eating disorder psychopathology, symptoms of food addiction and poor dieting success [20]. When compared with the longer, original tool, this reduced version has a strong level of internal consistency (Cronbach’s α = 0.94) and validity [20].

### 2.6. Data Analysis

Descriptive statistics are reported using mean ± standard deviation for symmetrically distributed variables and percentage for categorical variables. The distributions of all numeric variables were assessed for skewness using formal tests of normality and through visual inspection of histograms. Responses on the PSQI scale were recoded into two groups: PSQI ≥ 5 (poor sleepers) and PSQI < 5 (good sleepers). Differences in the measures of SHC and FCQ-T-r were tested using the Mann–Whitney U test. Spearman’s rank-order correlations were conducted to determine any relationships between variables. The effect size statistic employed for the Mann–Whitney test was *r*, which is the z value divided by the square root of the total number of observations [21]. Effect size was interpreted using the criteria, whereby 0.1 = small effect, 0.3 = medium effect and 0.5 = large effect [22]. In all cases, *p* < 0.05 was considered statistically significant.

## 3. Results

**Figure 1:**
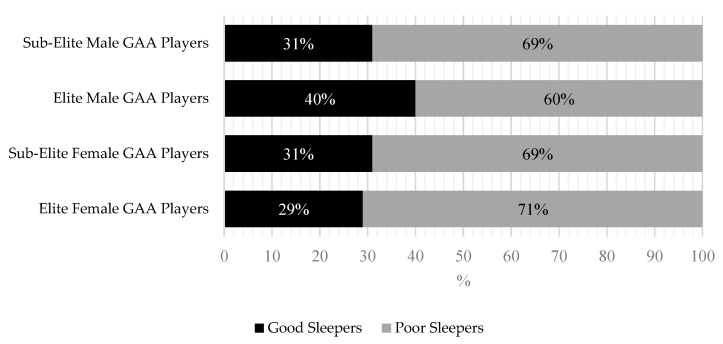
The proportion (%) of good sleepers to bad sleepers across each subcategory (sub-elite, elite, male and female GAA players).

**Figure 2:**
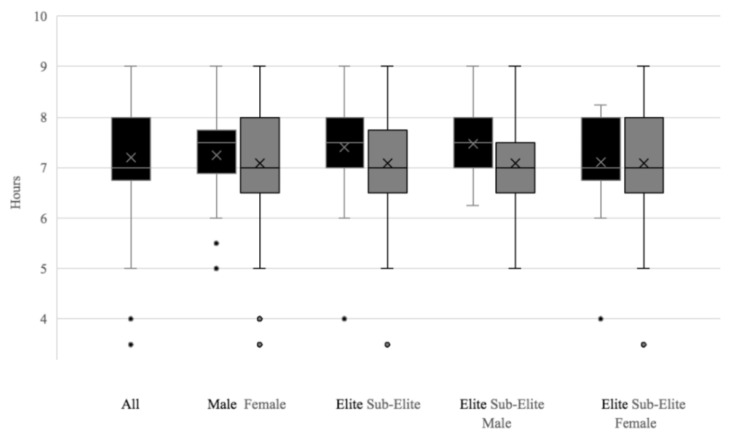
Sleep duration in hours (mean ± SD) as assessed by the PQSI across each subcategory (sub-elite, elite, male and female GAA players).

Participant characteristics are displayed in Table 1. The proportion of good sleepers and poor sleepers across sub-categories is displayed in Figure 1. The mean sleep duration for each subcategory is illustrated in Figure 2. There was no significant difference in BMI between good and poor sleepers (*p* > 0.05) A Spearman’s rank-order correlation found a very weak positive correlation between sleep and BMI; this was not statistically significant (rs = 0.087, *p* = 0.260).

### 3.1. PSQI Scores

There were no significant gender differences in PSQI scores (*p* = 0.088). Although, female players did have a higher mean PSQI score (6.71 ± 3.28) than male players (5.60 ± 2.34). Elite players had lower mean PSQI scores (5.44 ± 2.43) when compared to sub-elite players (6.32 ± 2.91). However, there were no significant differences between the groups (*p* = 0.072). In male athletes, there were no significant differences in PSQI scores between elite and sub-elite players (*p* = 0.052). However, elite players did have a lower mean PSQI score when compared to sub-elite players (Table 1). In female athletes, there were no significant differences across playing levels (*p* = 0.886).

#### 3.1.1. Sleep and General Health

**Figure 3:**
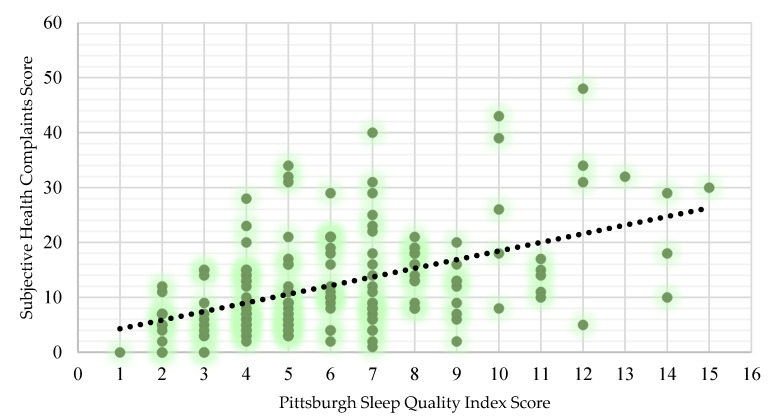
The statistically significant moderate, positive relationship between general health and sleep in GAA players (rs = 0.461, *p* < 0.001). The black circle line indicates a trend line. The trendline is included to show the pattern of the data between the two variables.

There was a significant difference in SHC between good sleepers and poor sleepers (U = 1829.5, *p* < 0.001). Poor sleepers (14.26 ± 9.78) were found to have higher SHC relative to good sleepers (7.93 ± 5.84), with a medium effect size (*r* = 0.35). There was a significant difference in SHC between males and females (U = 2161.5, *p* < 0.001). Females (15.51 ± 9.76) were found to have moderately higher SHC compared to males (10.11 ± 8.17; *r* = 0.31). There were no significant differences in SHC between elite and sub-elite players. A Spearman’s rank-order correlation found a statistically significant (rs = 0.461, *p* < 0.001) moderate, positive correlation between PSQI and SHC (Figure 3).

#### 3.1.2. Sleep and Food Cravings

**Figure 4:**
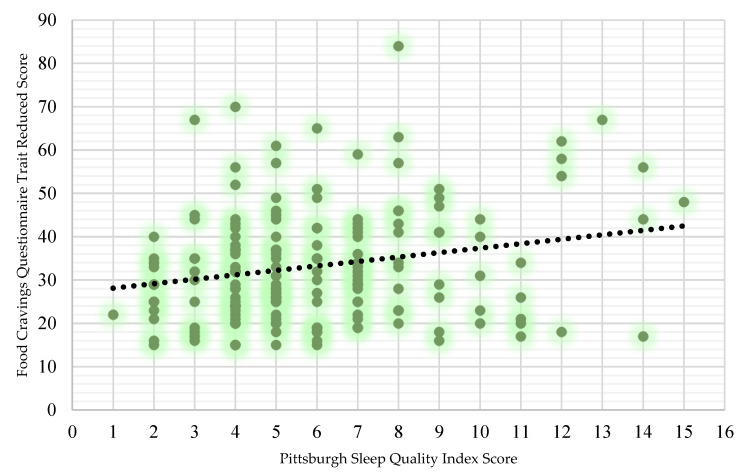
The statistically significant weak, positive relationship between food cravings and sleep in GAA players (rs = 0.174, *p* < 0.05). The black circle line indicates a trend line. The trendline is included to show the pattern of the data between the two variables.

There was a significant difference in food cravings between males and females (U = 2635, *p* = 0.013). Females (36.65 ± 14.39) were found to have higher food cravings when compared to males (31.18 ± 11.89; *r* = 0.19). Poor sleepers (34.44 ± 13.57) experienced higher FCQ-T-r scores on average than good sleepers (30.89 ± 11.96). However, no significant differences were reported (*p* = 0.104). There were no significant differences in food cravings between elite and sub-elite players (*p* = 0.68). A Spearman’s rank-order correlation found a weak, positive correlation between PSQI and food cravings, which was statistically significant (rs = 0.174, *p* < 0.05) (Figure 4).

## 4. Discussion

The aim of the present study was to (i) explore the sleep profile of both male and female GAA athletes, (ii) investigate the relationship between poor sleep and general health and (iii) food cravings. To date, very limited research exists examining the relationship between sleep, health and dietary behaviours.

### 4.1. Poor Sleep in GAA Athletes

Sixty-seven percent (*n* = 114) of GAA athletes in this study were categorised as poor sleepers. This falls within the range presented in a recent consensus statement which reported the presence of sleep disturbance in 50–78% of elite athletes [8]. However, this is notably higher than data in other intermittent team athletes: 48% of 68 elite male GAA athletes [5], 54% of 230 male and female college-level soccer athletes [23], and 50% of 175 elite rugby and cricket players [24]. Previous research has shown that team sport athletes are a cohort that tend to display worsened sleep characteristics [25,26]. It is also important to note that GAA players are amateur athletes balancing work and sport. Despite this, the sleep duration reported in elite male GAA athletes is almost identical to that of Biggins and colleagues [5], with players sleeping 7.5 ± 0.6 h in both studies. The most similar findings to ours come from a cohort of male (*n* = 113) and female (*n* = 24) amateur rugby players in Ireland, where 61% of players were classified as poor sleepers [27]. However, it is important to acknowledge that both studies only assessed athletes’ sleep during the pre-season [5,27]. In contrast, the present study encompasses a large proportion of competitive in-season athletes, a period where team sport athletes may obtain up to 30% less sleep on the night of games [28]. The present study has a relatively large sample of female (*n* = 65) participants, a cohort considered more likely to report poorer subjective sleep than their male counterparts [23]. This study presents a strong trend towards the presence of poor sleep in both males and females, elite and sub-elite GAA athletes.

There were no significant differences in PSQI between elite and sub-elite players. While this highlights the fact both cohorts may face challenging societal factors which may negatively impact sleep, our findings are somewhat in contrast to the literature. The prevalence of inadequate sleep has previously been suggested to be higher among elite athletic populations who often experience disruptive training and competition schedules which may limit the opportunity for optimal sleep [8]. Furthermore, the influence of a variety of sport-specific factors like high training loads and travel are thought to negatively impact sleep in elite athletes [8]. Indeed, it remains unclear whether a causal relationship exists between participation in elite sport and sleep inadequacy.

Elite male GAA athletes (*n* = 40) had the lowest proportion of poor sleepers (60%) to good sleepers (40%) across all sub-categories. This finding contrasted with elite female GAA athletes (*n* = 17) who had the highest proportion of poor sleepers (71%) to good sleepers (29%). These findings resemble those from a study investigating elite French athletes, where female athletes had a greater incidence of sleep problems and gender was identified as a risk factor for sleep problems [8]. Gender differences in elite sport have been highlighted, with differences in financial, promotional incentives and access to support having been observed. In addition, female athletes are often under-represented in scientific publications [29], and therefore future studies need to address this.

### 4.2. Poor Sleep and General Health

Insufficient sleep is associated with a variety of chronic illnesses and even premature mortality [30,31]. This study discovered that poor sleepers had significantly higher SHC compared to good sleepers in both male and female GAA athletes. In addition, a moderate, positive correlation was found between PSQI and SHC, namely, poor sleep and lower general health. Our findings build and expand on that of Biggins and colleagues [5], where elite male GAA athletes classified as poor sleepers had significantly increased SHC. Our findings are consistent with other literature which highlights poor sleep as a risk factor for compromised immune system function and increased susceptibility to infection [32,33]. In elite athletes, a case-controlled study reported that poor sleep was associated with a greater risk for upper respiratory tract and gastrointestinal symptoms [24]. Evidence has previously demonstrated a significant association between SHC and new injury risk in athletic populations; a study by Nobari and colleagues [34] involving 122 athletes discovered that <8 h of sleep per night increased the risk of injury by 65% [35]. While an explicit cause and effect cannot be determined from the nature of these findings, a clear reciprocal relationship between the variables is apparent. Interestingly, females were found to have significantly higher SHC than males. This is in line with the literature on Olympic athletes which discovered female athletes were at higher odds of illness [10]. Despite females making up nearly 50% of sports participants, they are often neglected in the literature with a mere 4% of research devoted to female-only cohorts [36]. They are often characterised as “more difficult” to study than men due to their level of hormonal intricacy [37]. This finding is the first to discover sex differences in the SHC of GAA athletes.

### 4.3. Poor Sleep and Food Cravings

There was a significant weak, positive correlation between PSQI and food cravings. Inadequate sleep is thought to stimulate a hedonic response which increases appetite, leading individuals to crave and consume excess amounts of highly palatable foods [38]. Despite poor sleepers experiencing higher mean FCQ-T-r scores than good sleepers, no significant differences were found. This is in contrast to the literature, which suggests that appetite is augmented under insufficient sleep and that eating habits are significantly associated with sleep [4,38]. However, one intervention study highlighted reduced food cravings as a result of moderate-intensity aerobic exercise [39]. Subjective sensations of hunger tend to diminish temporarily during high-intensity exercise and are commonly referred to as exercise-induced appetite suppression. Furthermore, current evidence suggests that physically active individuals have improved appetite sensitivity, which could generate long-term energy balance [40]. Perhaps the active nature of our cohort may have somewhat mitigated any effect poor sleep may have on food cravings. At the same time, there is no gold standard method of assessing food cravings and self-reported measures may be at risk of recall bias [38].

Interestingly, females were found to have significantly higher food cravings compared to males. Little evidence pertaining to gender differences in athlete food cravings exists. However, these findings are in line with the literature which suggests that women report experiencing stronger trait food cravings than men [41]. It is thought to be a complex interaction of biological, sociological and environmental factors that account for such gender differences in food cravings [41]. This finding may also be linked with the intentional reduction in food intake and increased prevalence of disordered eating that has been previously demonstrated amongst female athletes [42]. In females, food consumption also fluctuates across the menstrual cycle, such that those in the luteal phase consume significantly more food and prefer sweeter foods compared to women in the follicular phase [43]. This appears to be attributed to the fluctuation of sex hormones, which are important modulators of food consumption [44]. In addition, research has revealed that the menstrual cycle influences sleep duration and this is also closely tied to hormonal changes, particularly the notable increase in progesterone levels during the luteal phase. Beyond sleep duration, the menstrual cycle can also impact sleep architecture, including various sleep stages (rapid eye movement (REM) and non-REM) ultimately influencing overall sleep quality. In turn, sleep quality might favour unhealthy food cravings. One potential mechanism may be that insufficient continuous sleep detrimentally affects brain development including the prefrontal cortex, which controls food cravings [45]. While nothing conclusive can be drawn from the present results, our findings support the idea that female GAA athletes may benefit from nutrition education programs [46]. Previous work has shown that this group have poor nutritional knowledge, not to mention a high prevalence of low energy availability, both of which may negatively impact dietary behaviour [36,47].

### 4.4. Limitations

The present study is the first to explore the relationship between sleep, general health and food cravings in both male and female GAA athletes, at an elite and sub-elite level. Despite this, it must be acknowledged that our research has a few limitations, and it is plausible that a number of them could have influenced the results obtained. Therefore, results from our analyses should be treated with prudence. It must be acknowledged that empirical debate surrounding the appropriate PSQI cut-off for athletes remains ongoing [48]. The PSQI is a subjective tool designed for assessing sleep in the general population, not specifically for sleep screening elite athlete populations [49]. As a result, this may suggest a possible overestimation of sleep problems in athletes and perhaps the Athlete Sleep Screening Questionnaire would be more suitable [3]. However, considering that athletes often strive for even the slightest improvements in performance the identification of ‘poor’ sleep at the standard cut-off of (≥5) is warranted [47]. The large recall period and retrospective nature of our measures may be subject to bias [50]. The convenience sample obtained may not be completely representative of the entire population of GAA athletes [8]; in addition, the different stages of the training cycle (in competition vs. off-season vs. pre-season) may have influenced some of the outcome measures as research has shown that sleep patterns and susceptibility to acute and recurrent infections may be altered during competition [8,51]. Specifically, the small sample size of highly trained female athletes warrants caution. Finally, dietary behaviour may be better represented by employing more commonly used dietary assessment methods from sports nutrition research [52].

### 4.5. Practical Applications and Future Research

The high prevalence of poor sleepers in the current study highlights the need for research-informed sleep education in the GAA. Frequent sleep education workshops along with frequent check-ins with the athlete about their sleep have been shown to increase sleep duration in athletes [8]. Our findings suggest that sleep-screening athletes may prove prudent in identifying and managing those most at risk of lower general health [8]. Female athletes have been largely neglected in the literature and future research should be prioritised in this population [36]. Finally, methods of assessment such as a food record (FR) or a combined dietary data collection method like the FR and 24 h dietary recall would allow for the quantification of energy intake and may offer a more comprehensive representation of dietary behaviour in GAA athletes [52]. In addition, future research should include more objective measures for assessing sleep quality (e.g., polysomnography, actigraphy), alongside self-reported measurements.

## 5. Conclusions

This study has gone some way towards enhancing our understanding of the relationship between sleep, general health and food cravings in GAA athletes. Sixty-seven percent of GAA athletes in the present study were found to report poor sleep. Given that sleep is an essential component of recovery in GAA athletes, sleep screening, education and intervention to enhance sleep is advocated. Results from this study indicate that a significant relationship exists between both poor sleep and lower general health as well as poor sleep and increased food cravings. Furthermore, our findings suggest that female GAA athletes may be at an increased risk of food cravings, lower general health and poorer sleep than their male counterparts.

## Figures and Tables

**Table 1:** Participant Characteristics across each subcategory (*n* = 170).

			Elite (*n* = 40)			Sub-Elite (*n* = 65)	
		Mean	SD	Range	Mean	SD	Range
Male (*n* = 105)	Age	25.48	6.28	18–41	24.03	4.39	18–36
	Height (cm)	180.25	8.17	155–206	181.65	7.53	145–200
	Weight (kg)	83.18	7.35	72–102	83.54	12.01	68–145
	BMI (kg/m^2^)	25.65	2.03	23–31	25.32	3.08	21–40
	Global PSQI Score	4.95	1.88	1–9	6	2.52	2–12
	SHC	8.82	5.98	0–23	10.91	9.21	0–48
	FCQ-T-r	30.13	12.02	15–67	31.83	11.85	15–62
			**Elite (*n* = 17)**			**Sub-Elite (*n* = 48)**	
		**Mean**	**SD**	**Range**	**Mean**	**SD**	**Range**
Female (*n* = 65)	Age	25.71	6.04	19–36	23.42	5.06	18–37
	Height (cm)	167.69	6.02	151–178	165.47	5.15	155–179
	Weight (kg)	68	8.44	55–85	63.37	7.84	47–92
	BMI (kg/m^2^)	24.13	3.61	20–30	23.1	2.7	19–34
	Global PSQI Score	6.59	3.18	3–14	6.75	3.34	2–15
	SHC	16.88	8.62	3–32	15.02	10.18	0–43
	FCQ-T-r	39	14.88	17–70	35.81	14.28	18–84

cm = Centimetres, kg = Kilograms, BMI = Body Mass Index, PSQI = Pittsburgh Sleep Quality Index (good sleepers < 5 and poor sleepers ≥ 5), SHC = Subjective Health Complaints, FCQ-T-r = Food Cravings Questionnaire-Trait-Reduced, SD = Standard Deviation.

## Data Availability

The data presented in this study are available on request from the corresponding author.
